# Prevalence of human parainfluenza virus in patients with acute respiratory tract infections in Beijing, 2011–2014

**DOI:** 10.1111/irv.12336

**Published:** 2015-10-13

**Authors:** Weixian Shi, Shujuan Cui, Cheng Gong, Tiegang Zhang, Xiali Yu, Aihua Li, Meng Chen, Ming Luo, Fang Huang

**Affiliations:** Beijing Center for Disease Control and PreventionBeijing, China

**Keywords:** Beijing, epidemiology, parainfluenza virus

## To the editor:

Human parainfluenza viruses (HPIVs) are one of the most frequent pathogens that circulate worldwide^1^. Four types of HPIVs were identified, including HPIV-1 to -4. HPIVs can lead to acute respiratory infection in young,^2^ the immunocompromised,^3^ and the elderly patients.^4^ The epidemiological character of parainfluenza virus in Beijing has not been previously reported.

In a consecutive 4-year surveillance of acute respiratory infections (ARIs) between January 2011 and December 2014, a total of 3978 specimens were collected from the fever outpatients with ARIs (with acute fever (temperature of ≥38°C) and cough or sore throat fewer than 5 days) by throat swab and sputum for those also with abnormal chest X-ray. The patients with ages ranging from 1 month to 96 years old (mean ± SD: 29·92 ± 23·13) were from five sentinel hospitals in Beijing, which covered about 8·6 million population of the city. The study was approved by the Ethical Committee of Beijing Center for Disease Prevention and Control (CDC). All samples were analyzed anonymously.

HPIV-1 to -4, and other respiratory viruses were analyzed by multiplex real-time PCR.^5^ RNA was extracted from samples using Qiagen QIAmp Viral RNA mini kit (QIAGEN, Hilden, Germany) and eluted in 60 μl elution buffer. The multiplex combined real-time PCR detection kit for respiratory viruses was purchased from Jiangsu Uninovo Biological Technology Co., LTD (Uninovo, Zhenjiang, China). HPIV-1 to -4 as well as influenza A and B virus, human bocavirus, rhinovirus, respiratory syncytial virus, enterovirus, adenovirus, human coronavirus, metapneumovirus were detected by four sets.

HPIVs were confirmed in 217 (5·46%) of 3978 specimens, including 56 HPIV-1 (25·81%), 33 HPIV-2 (15·21%), 88 HPIV-3 (40·55%), and 40 HPIV-4 (18·43%). Other respiratory viruses detected were 488 (12·27%) influenza viruses, 191 (4·80%) rhinoviruses, 92 (2·31%) respiratory syncytial viruses, 88 (2·21%) enteroviruses, 70 (1·76%) adenoviruses, 62 (1·56%) human bocaviruses, 35 (0·88%) human coronaviruses, and 33 (0·83%) metapneumoviruses.

**Figure 1:**
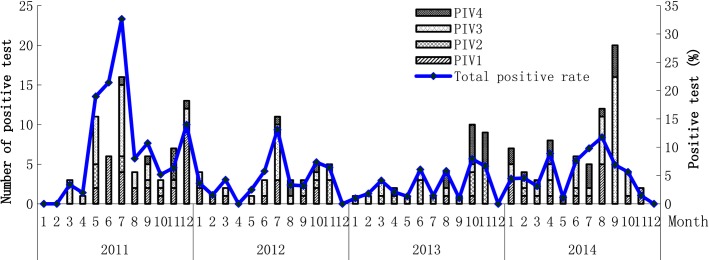
Detection results of hunman parainfluenza viruses in different months during 2011–2014 in Beijing.

Although HPIV-positive patients were detected in 125 (5·65%) of the 2214 males and in 92 (5·22%) of the 1764 females, the HPIV-positive ratio of males and females had no significant difference (χ^2^ = 0·353, *P* = 0·574). All types of HPIVs were detected every year, and the HPIVs-positive rates in 2011 (70/772, 9·07%) and in 2014 (72/1304, 5·52%) were significantly higher than those in 2012 (38/850, 4·47%) (χ^2^ = 13·75, *P* < 0·001) and 2013 (37/1052, 3·52%) (χ^2^ = 24·84, *P* < 0·001). The positive rates of HPIV-1 and HPIV-3 declined year by year during 2011–2013 and increased in 2014, while the positive rate of HPIV-2 showed a biennial trend and there were no obvious annual trend for HPIV-4 (Table1). The data of surveillance also indicated that the HPIV-positive rates were significantly higher during June to August than those in other months (χ^2^ = 18·79, *P* < 0·001). The seasonal trend showed an increase month by month from May to the peak by July and then kept going down rest of months. Interestingly, there was a small peak during October and December in almost every year. Although HPIV-1 and HPIV-3 showed the same seasonal trend mentioned above, HPIV-3 was shown a longer spread than HPIV-1. High peaks of HPIV-2 were seen in April/May/June/July/October/November, and the HPIV-4-positive rates were higher during July to November (Figure1). HPIVs attack population of all ages. The highest rates of age-specific infection, however, were observed in children under 5-year-old group (67/753, 7·75%) and followed by the patients over 60-year-old group (37/678, 5·49%). The teenage group (6- to 15-year-old group) had the lowest positive rate (21/519, 4·05%). The differences in age-specific positive rates were significant (χ^2^ = 26·168, *P* < 0·001). HPIV-3 was the main type for the children under 5-year-old group (23/39, 58·97%).

**Table 1:** Clinical characteristics of patients with HPIV infections [*n* (%)]

Parameters	No. of sample	HPIV	HPIV-1	HPIV-2	HPIV-3	HPIV-4
Year						
2011	772	70 (9·07)	28 (40·00)	8 (11·43)	27 (38·57)	7 (10·00)
2012	850	38 (4·47)	11 (28·95)	5 (13·16)	17 (44·74)	5 (13·16)
2013	1052	37 (3·52)	2 (5·40)	15 (40·54)	8 (21·62)	12 (32·43)
2014	1304	72 (5·52)	15 (20·83)	5 (6·94)	36 (50·00)	16 (22·22)
Total	3978	217 (5·46)	56 (25·81)	33 (15·21)	88 (40·55)	40 (18·43)
Gender						
Male	2214	125 (5·65)	34 (27·20)	19 (15·2)	57 (45·60)	15 (12·00)
Female	1764	92 (5·22)	22 (23·91)	14 (15·22)	31 (33·69)	25 (27·17)
Age group (Years)						
0–5	753	67 (7·75)	8 (11·94)	12 (17·91)	37 (55·22)	10 (14·93)
6–15	519	21 (4·05)	5 (23·81)	4 (19·05)	7 (33·33)	5 (23·81)
16–25	615	26 (4·23)	12 (46·15)	2 (7·67)	7 (26·92)	5 (19·23)
26–59	1413	66 (4·67)	22 (33·333)	6 (9·09)	22 (33·33)	16 (24·24)
≥60	678	37 (5·46)	9 (24·32)	9 (24·32)	15 (40·54)	4 (10·81)
Infection sites						
URTIs*	2171	127 (5·85)	33 (25·98)	21 (16·54)	42 (33·07)	30 (23·62)
LRTIs**	1807	90 (4·98)	23 (25·56)	12 (13·33)	46 (51·11)	10 (11·11)

* Upper respiratory tract infection.

** Lower respiratory tract infection.

Of the 217 HPIVs-positive specimens, 63 (29·03%) were positive for other respiratory viruses simultaneously. The codetection occurred mainly by human rhinovirus (21/63, 33·33%) and followed by enterovirus and influenza virus (12/63, 19·05%; 10/63, 15·87%). The majority of codetection was linked to HPIV-3 39·68% (25/63,) and HPIV-1 25·40% (16/63), HPIV-4 20·63% (13/63), HPIV-2 14·29% (9/63), respectively. Of the 3978 patients, 90 in 1807 with lower respiratory tract infection (4·98%) and 127 in 2171 with upper respiratory tract infection (5·85%) were HPIV positive. There was no significant difference between the different clinical diagnosis (χ^2^ = 1·45, *P* = 0·234). All the patients infected by HPIVs had fever with a similar maximum body temperature (mean ± SD): HPIV-1 (38·4 ± 0·8), HPIV-2 (38·4 ± 0·9), HPIV-3 (38·4 ± 0·5), and HPIV-4 (38·4 ± 0·6). Other symptoms frequently encountered included cough (69·79%), expectoration (38·54%), sore throat (33·33%), sneezing (32·29%), asthenia (31·25%), headache (26·04%), normal leukocyte count (84·35%), low leukocyte count (3·12%), normal lymphocyte count (95·83%), with radiological pulmonary abnormalities (29·17%).

This 4-year consecutive study reported the prevalence and basic clinical features of HPIVs infections in patients with symptoms of ARIs during 2011-2014. HPIV-3 (88/217, 40·55%) and HPIV-1 (569/217, 25·81%) led to the most prevalent HPIVs infection in Beijing. In accordance with our results, a study performed in Yamagata, Japan, between 2002 and 2011 also showed that HPIV-3 was the main HPIV causative agent of ARIs in children.^6^ But because wider age-group patients were considered, our results showed that infants, young children, and elderly were the risk populations of HPIVs infection, especially HPIV-3 infection. The higher infections by HPIVs in summer in Beijing and the biennial trend for HPIV-2 were consistent with other reports.^2^,^6^ The circulating types and related intensity varied in different year, and the type-specific seasonal patterns were not well understood. Whether the higher detection rate in 2011 and 2014 was from the naturally rising HPIVs infection in population or from the different epidemiological trend for different types of HPIVs, more surveillance data would be needed. Our study also showed that the HPIVs were the secondly main respiratory pathogens for ARIs patients, so clarifying the epidemiology of the HPIVs is important for understanding and providing information to evaluate the respiratory disease burden in clinics and disease control policies.
